# Quantification of the Influence of Concrete Width per Fiber Strand on the Splitting Crack Failure of Textile Reinforced Concrete (TRC)

**DOI:** 10.3390/polym14030489

**Published:** 2022-01-26

**Authors:** Markus Beßling, Jeanette Orlowsky

**Affiliations:** Department of Building Materials, TU Dortmund University, 44227 Dortmund, Germany; jeanette.orlowsky@tu-dortmund.de

**Keywords:** TRC, splitting failure, influence of the concrete, model

## Abstract

The composite material textile reinforced concrete (TRC) requires a high bond performance between the fiber strand and the concrete matrix. While the influence of the textile on bond behavior is well known, in this publication the influence of the concrete matrix is investigated by means of single-sided pull-out tests. The results of the presented study show dependence between the concrete strength and bond performance of the composite material. When a concrete of a higher-strength class is used, the bond-flow–pull-out distance curve shifts upward independent of the textile geometry and the yarn impregnation. A simplified model is presented to predict the occurrence of a crack along the fiber strand. This model serves as a basis to investigate the correlation between concrete width per fiber strand and resistance against a splitting crack. The effective concrete tensile strength decreases to about 35% when the concrete width is increased from 10 mm to 50 mm. To quantify the decrease, a mathematical relationship, which describes the test results independent of textile and concrete type, is proposed.

## 1. Introduction

The composite material textile reinforced concrete (TRC), which is composed of a textile reinforcement and a concrete matrix with a small maximum grain size, is increasingly establishing itself in the construction industry. The use of TRC makes sense both in new construction and in repair work [1,2,3,4]. A great advantage of TRC compared to conventional steel reinforced concrete is the material saving, which results in a reduction of weight and consumed resources [5]. Due to the high durability of carbon fibers, the concrete cover can be reduced to a minimum necessary for the bond. Consequently, the construction of slender component geometries is possible.

**Figure 1:**
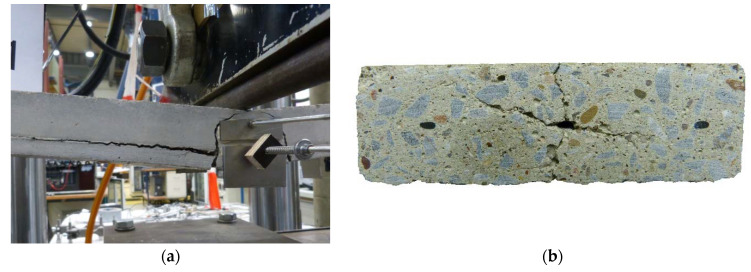
(**a**) Splitting failure of a TRC component tested in a bending tensile test; (**b**) cracking parallel and orthogonal to the textile layer in a cross section of an anchorage length test specimen (photos: Beßling).

In addition to the high tensile strength of carbon fibers, the geometry of textile reinforcement combined with a stiff impregnation can lead to high bond performance. Thus, fine crack patterns and short anchorage lengths can be realized. However, large bond forces also place greater stress on the surrounding concrete and thus increase the risk of cracking along the fiber strand (see Figure 1). If the transversal forces resulting from the bond exceed the resistance forces of the concrete matrix, cracking will occur along the fiber strand. In components made of TRC, failure can therefore often be observed due to spalling of the concrete cover and/or longitudinal cracking (see Figure 1). The cracks not only impair the visual appearance but also represent a decisive type of failure, especially in the end-anchorage and overlap areas [6,7,8,9].

**Figure 2:**
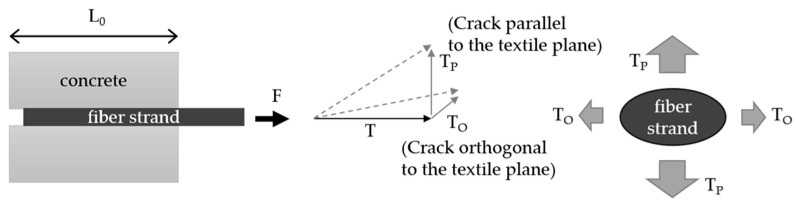
Terms for describing the orthogonal and parallel forces along the fiber strand.

When a fiber strand is pulled out of the concrete, shear forces on the outside of the strand counteract the pull-out. With the pull-out force at the end of the yarn (F) related to the bond length of the fiber strand (L_0_), a bond flow T = F/L_0_ (N/mm) can be calculated (Figure 2). Bond forces can be transmitted via the mechanisms of adhesive bond, frictional bond, and form fit [8,10,11]. The bond flow can be influenced by various textile parameters. For example, the impregnation material, which crosslinks the individual fibers within the fiber strand, has a significant influence on the bond forces. The stiffer the material, the greater the bond forces that can be transmitted [12,13,14]. The textile geometry (e.g., fiber strand-geometry and weave type) also influences the bond forces [7]. In this context, the textile processing creates yarn structures that can be similar to the ribbing of reinforcing steel bars.

If mineral-based impregnations are used, an adhesive bond can additionally be realized between the fiber strand and the concrete matrix [15]. This ensures a comparatively high initial bond and allows for a fine crack pattern and small crack spacing [16]. Additionally, the reduction of the bond performance due to high temperatures can clearly be reduced [15].

Various test setups can be used to determine the magnitude of the bond flow [14,15,17,18]. Thus, a defined length (L_0_) of the fiber strand is pulled out of the concrete and the pull-out distance is measured. The result is a bond-flow–pull-out distance (crack opening) curve. This is necessary to calculate a bond-stress–slip relationship [11], which provides the basis for the computational determination of crack spacing, anchorage and overlap lengths. These properties can also be determined with experiments. In [6,8,14,16], the different test setups are presented. By using a single-sided pull-out test setup with a locally reduced concrete width, the splitting tendency of a single fiber strand related to a bond length (L_0_) of one spacing of the nodes can be investigated [8,19].

The bond forces along the fiber strand also take effect in the boundary zone to the concrete. In the concrete matrix, the load transfer can be represented in a simplified way with forces orthogonal (T_P_) and parallel (T_O_) to the fiber strand (see Figure 2), respectively textile plane [14,19,20].

**Figure 3:**
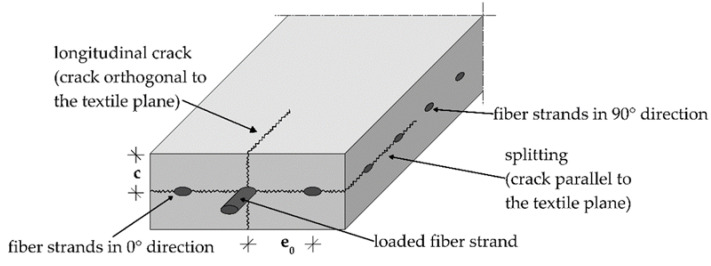
Overview of the types of cracking along a fiber strand of a textile.

In [7,19,20,21], models for the calculation of crack formation along the fiber strand are presented. They focus on the textile processing and the associated geometric properties of the yarn. Preinstorfer [22] shows that the cross-sectional shape of the yarn in combination with its change along the yarn length significantly influences the direction and magnitude of the resulting transversal forces (T_O_ and T_P_) in the concrete. The ribs of the textile fiber strands have a smaller inclination angle compared to the ribbing of the reinforcing steel. This means that the angle of the compression strut inclination and thus the resulting transversal forces are relatively larger [8]. If the acting forces exceed the forces that can be absorbed in the concrete, cracking occurs along the fiber strand. Depending on the mesh width, the yarn cross-section shape, and the concrete cover c, splitting cracks and/or longitudinal cracks develop. Figure 3 shows an overview of the crack types. The fiber-strand spacing (e_0_ = fiber strand center to center distance in 0° direction) can significantly influence the load-bearing behavior of the composite. Thus, the tensile load-bearing capacity, the anchorage length, the crack spacing, and the crack widths can be specifically controlled. The textile processing allows for stepwise variation of the fiber-strand spacing.

**Figure 4:**
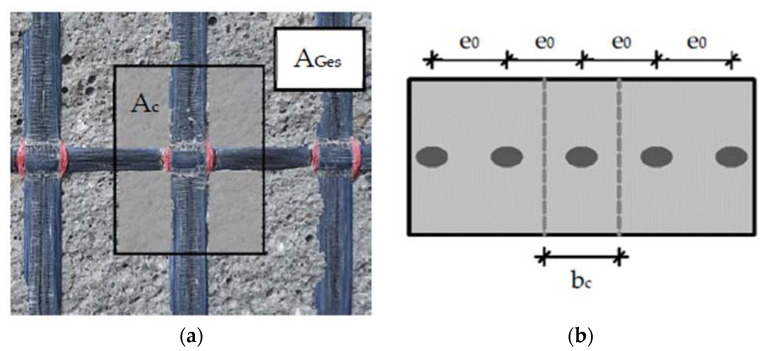
(**a**) Effective concrete area in the textile plane (A_c_) per fiber strand and total area in the textile plane per fiber strand (A_Ges_ = A_c_ + A_textile_); (**b**) the concrete width per fiber strand (b_c_) is equal to the center-to-center distance of the fiber strands (e_0_).

While the textile properties primarily influence the “action side” with regard to possible cracking, the concrete properties mainly determine the “resistance side”. The resistance is calculated from the tensile strength of the concrete and the effective concrete area per fiber strand perpendicular or parallel to the textile plane. Ortlepp [23] concluded that the effectively existing concrete area preventing a splitting crack can be calculated by a covering factor k_A,eff_ = A_c_/A_Ges_ (A_c_ = area of the concrete matrix, A_Ges_ = total area = area of the concrete matrix and area of the textile) of the textile (see Figure 4a).

In [22], an additional reduction factor for the concrete tensile strength is applied, which considers the stress distribution within the concrete. In addition to the tests with a normal concrete strength (f_c,m_ = 75.2), one test series was performed using a UHPC (f_c,m_ = 137.3). It is shown that the bond-slip curves for both concretes are similar and the maximum load in case of failure due to a splitting crack is only slightly increased with the UHPC. This is justified by the comparatively higher brittleness of the UHPC.

While previous investigations have mainly focused on the properties of the textile and the associated effect on cracking along the fiber strand, the influence of the concrete matrix will be considered in more detail in this publication. The aim of the investigations is to evaluate the influence of the concrete on the undisturbed (without cracking along the fiber strand) bond performance of different textiles. Furthermore, the influence of the effective concrete width on the resistance against splitting is investigated.

## 2. Materials and Methods

### 2.1. Materials

**Figure 5:**
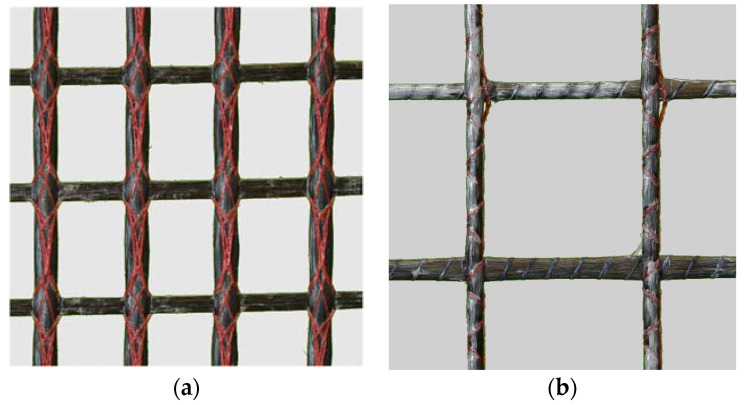
Geometries of the used textiles (**a**) geometry 1 (T1); (**b**) geometry 2 (T2).

The tests were carried out with four different textiles made by V. FRAAS Solutions in Textile GmbH (Now WILHELM KNEITZ SOLUTIONS IN TEXTILE GMBH). These vary in mesh size, impregnation agent, and fiber-strand geometry. Geometry 1 (see Figure 5a) has a mesh size of 12.7 × 16.0 mm^2^, while geometry 2 has a larger mesh size. The two textiles also differ in the geometry of the fiber strand. Textile 1 is characterized by a single expansion between the nodes, while the second textile has multiple expansions between the nodes.

Both textiles have a 48k carbon roving in the 0° direction, which has a flat cross-sectional shape in textile 1 and is approximately round in textile 2. Textile 1 will be investigated in two impregnation variants. The acrylate impregnation provides a significantly higher stiffness compared to the impregnation with SBR (styrene butadiene rubber). Textile T1A and T1bA differ in terms of the widening of the fiber strand between the junction points. In Table 1, the properties of the used textiles are summarized.

Due to the smaller mesh size and the flat yarn cross-section, textile 1 has a lower covering factor (determined on the basis of Ortlepp [23]), which describes the ratio of the uncovered concrete area to the total area. This factor is later used for calculating the effective concrete width, which counteracts cracking parallel to the textile plane.

Two prefabricated fine-grained concrete mixes from PAGEL Spezial-Beton GmbH and Co. KG are used (C1 and C2). Both mixtures have a maximum grain size of 1 mm. The properties of the hardened concrete according to EN 196 (storage: 1d covered in the formwork, 6d water bath, 21 d 20 °C/65% rel. hum) are presented in Table 2. Compared to C1, the compressive and flexural strength of fine-grained concrete C2 is about 30% higher. The E-modulus of C2 is also higher. To determine the factor δ, which describes the ratio of centric tensile strength to flexural tensile strength, centric tensile tests were performed on cores with a diameter of 50 mm. The cores were taken from unreinforced specimens in order to consider the influences of specimen production.

### 2.2. Experimental Setup and Specimen Fabrication

**Figure 6:**
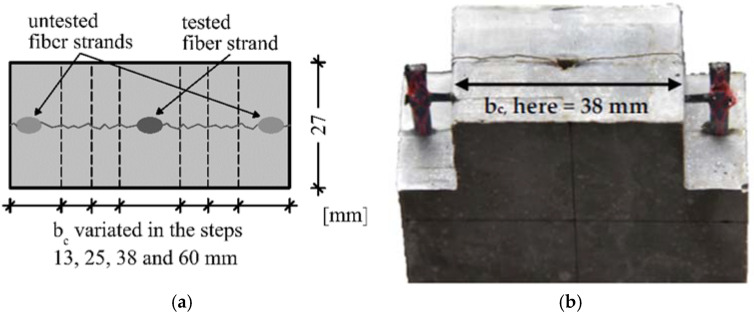
(**a**) Variation of the concrete width (b_c_); (**b**) implementation in the specimen (here b_c_ = 38 mm).

The studies are executed on single-sided pull-out tests with different concrete widths (b_c_). The value varies between 13 mm and 60 mm (see Figure 6). The concrete width bc in the specimen corresponds to the center distance of the fiber strands (e_0_) (see Figure 4b) of a textile in a component. To determine the undisturbed bond performance, tests are carried out on the full cross-section (b_c_ = 60 mm). In order to investigate the influence of several fiber strands that are next to each other, tests are also carried out on three tested fiber strands (y3 series, b_c_ = 13 mm). 

To increase the significance of the results, the experiments were performed with two fine-grained concrete mixes (C1 and C2) and four different textiles. The textiles differ in yarn geometry, impregnating agent, and mesh size.

**Figure 7:**
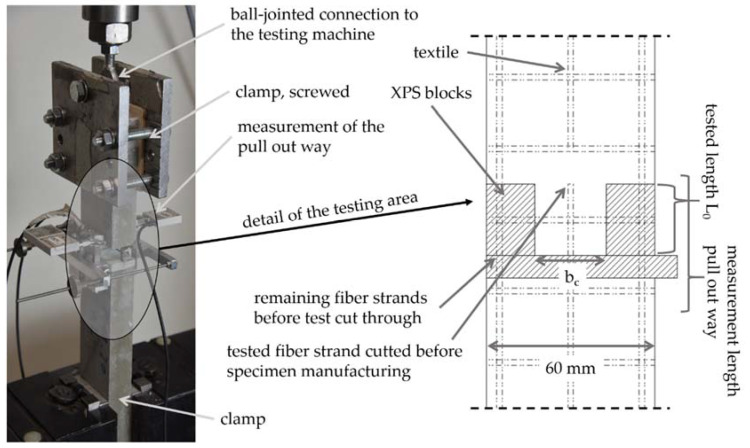
Test setup (**left**) and detail of the tapered area and the predetermined breaking point (**right**).

For the investigations, single-sided pull-out tests based on [19] are carried out. The test specimens are divided into an upper part, which contains the fiber strand to be tested, and a lower part, which ensures the anchoring of the fiber strand. Both parts are connected to each other via a predetermined breaking point. This is accomplished by means of embedded blocks made of extruded polystyrene foam (XPS) (see Figure 7 right). Thus, the parts of the specimen are completely separated from each other and no initial crack occurs in the concrete (compare [14]). XPS blocks are also used to produce the taper of the concrete cross-section. A smooth adhesive tape minimizes the bond between the XPS and the concrete. To ensure high positional stability of the textile and precise setting of the concrete width (b_c_), the test specimens are produced upright in steel formworks using a casting process. The anchorage length of the fiber strand (L_0_) is limited by a separating cut before concreting. After production, the specimens are left in the formwork for 1 day, followed by water immersion for 6 d and storage in a climatic chamber at 20 °C and 65% relative humidity until testing after 28 d. To determine the concrete properties, one set of prisms per manufacturing charge is prepared according to EN 196 and also tested on the day of the test. Six specimens are produced and tested for each test series. One manufacturing charge contains one or two testing series. All specimens had thickness of t = 20 mm.

For testing, the lower part of the specimen is clamped directly with a clamping device, while the upper part of the specimen is screwed between two steel plates and connected to the testing machine with a ball joint. After applying a preload, the lateral XPS blocks are removed from the specimen and the remaining fiber strands are cut. Then, the equipment for measuring the pull-out distance is set up (three DD1 displacement transducers from HBM). The pull-out distance of the fiber strand in the center of the specimen can thus be calculated via triangulation. A possible tilting of the specimen head can also be evaluated via the measurement.

The test is carried out until the fiber strand is completely pulled out, whereby the measuring equipment is removed in advance for protection. After the test, the anchorage length of the test yarn L_0_ and the concrete width (b_c_) are determined. The failure modes observed are pullout without cracking or pull-out with a splitting crack (compare Figure 5b). Only if splitting of the specimen occurs is an evaluation of the splitting tendency is possible.

### 2.3. Model to Predict Cracking along the Fiber Strand in TRC

**Figure 8:**
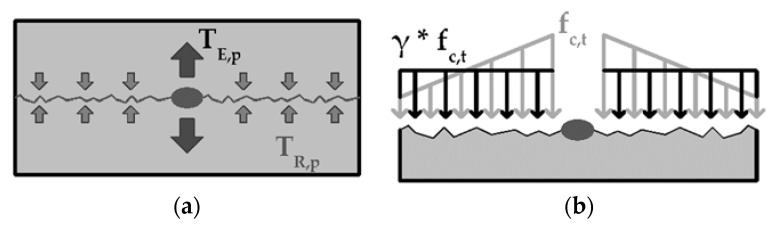
(**a**) Acting (T_E,p_) and reacting (T_R,p_) forces along a crack parallel to the textile plane; (**b**) transfer of the reacting concrete stresses into a stress block.

The investigations on the influence of the concrete width on cracking along the fiber strand are analyzed with a simplified mechanical model based on the work of Lorenz et al. [20] and Preinstorfer [22] (see Figure 8). The action side (T_E,p_) is calculated via the currently acting bond flow (T) multiplied by a splitting tendency factor α_P_. The factor α_P_ describes the ratio between T_P_/T and is determined on the basis of test results. The factor is a textile-specific parameter.

The acting force (T_E,p_) is counteracted by the concrete matrix. The maximum force that can be absorbed by the matrix (T_R,p_) can be calculated by multiplying the tensile strength (f_c,t_) of the concrete by the effective concrete width (e_c,0_). This is calculated by multiplying the covering factor k_a,eff_ with the fiber strand distance e_0_. Furthermore, it is assumed that the stresses in the concrete are not constant over the full width but decrease with increasing distance to the fiber strand (compare [22]). To quantify this influence, factor γ is introduced into the calculation. The factor describes the conversion of the actual stresses into a stress block with the same area. This is determined as a function of the mesh width e_0,_ respectively, the concrete width per fiber strand b_c_.

The equilibrium of forces in the cross-section can be described as follows:

(1) $$\propto_{p}*T=T_{E,p}=T_{R,p}=f_{c,t}*e_{c,0}*\gamma$$

with:

*α_p_*: splitting tendency factor of the fiber strand
*T*: actual bond flow
$f_{c,t}=\delta *f_{c,fl}$: centric tensile strength of the concrete

with

*δ*: conversion factor centric tensile strength/flexural tensile strength
*f_c,fl_*: flexural tensile strength of the respective manufacturing charge
$e_{c,0}=\frac{A_{c}}{A_{Ges}}*e_{0}$: effective concrete width

with

*e*_0_: center to center distance of 0° fiber strands (see Figure 4b)
*e*_0_ = b_c_: concrete width per fiber strand in case of test specimen
$\gamma =f\left(e_{0}\right)$: conversion factor for stress distribution

## 3. Results

### 3.1. Influence of the Concrete Properties on the Bond-Flow–Pull-Out Way-Curves 

**Figure 9:**
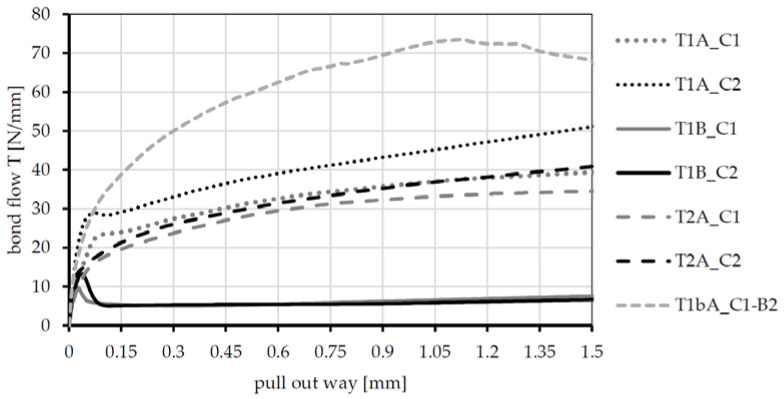
Bond-flow (T)—pull-out way-curves of the four textiles embedded in concrete C1 and C2 carried out on specimens with a width of b_c_ = 60 mm, with mean value curves of six single value curves each (exception T1bA: one representative curve).

The tests on the specimens with a concrete width of 60 mm allow for a comparison of the bond performance of the different textiles in combination with the two concretes. Figure 9 shows the bond flow–pull-out way-curves. The curves represent mean value curves, which were determined from six normalized single value curves each. Only in case of textile T1bA, a representative single curve is shown. In these tests, splitting of the specimens already occurred in the range up to 1.5 mm pull-out distance, and the mean value curve would thus be distorted.

A comparison of the textiles confirms the existing findings on the influence of the impregnation and the geometric configuration of the fiber strand. Textile T1B (SBR impregnated) achieves the lowest bond forces and shows the typical curve of a soft impregnated textile with the curve section’s adhesive bond, destruction of the adhesive bond, and frictional bond. Textile T1A has a similar geometry but is impregnated with a much stiffer agent (acrylate). This significantly increases the bond forces. Additional to the frictional bond, forces can also be transmitted via form fit. With the same impregnating agent but more pronounced waviness between the nodes (textile T1bA), the bond curve shifts further upward. The lower waviness of the T2 textile compared to the T1 textile results in a somewhat lower bonding performance compared to T1A.

The influence of the concrete can be observed by comparing the curves for each textile type (compare grey and dark curves). It can be seen that the higher-strength concrete (C2) leads to an increase in bond performance, irrespective of the type of textile. In this case, the entire bond curve shifts upward. This can be explained by the higher density of the concrete structure and the associated reduced porosity, as well as an increase in the transverse strain stiffness. Compared to the influence of the textile parameters, however, the influence of the concrete is much smaller (compare T1B, T1A, and T1bA for C1). For further analyses, it can therefore be assumed that concrete C2 allows for a slightly higher bond performance compared to C1.

### 3.2. Determination of the Splitting-Tendency factors (α_p_) of the Textiles

**Figure 10:**
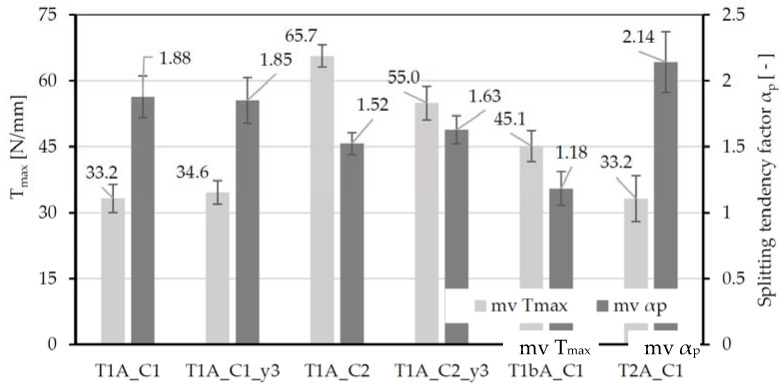
Overview of the reached maximum bond flows (T_max_) and the associated splitting-tendency factors for b_c_ = 13 mm, mv = mean value, and standard deviation (sd) given as error indicator.

The α_p_ (=T_p_/T) factor presented in 2.4 indicates the relationship between the currently acting bond flow (T) and the resulting force flow orthogonal to the textile plane (T_p_). A larger α_p_ value thus implies a higher splitting tendency of the textile. The value is determined using the tests with the smallest concrete width (b_c_ = 13 mm). The basis for the determination is the assumption that the splitting crack occurs at the moment of maximum bond flow, which is shown in [26]. Furthermore, the factor γ for the stress distribution in the concrete is set equal to 1.0. It is assumed that for a concrete width (b_c_) of <13 mm, the stresses can be considered constant in a simplified way. On this basis, the α_p_ factors for the different textiles in combination with the two concretes can be determined. Figure 10 shows an overview of the maximum bond flows T_max_ as well as the splitting-tendency factors α_p_.

The results show, that th e influence of the number of tested fiber strands is minor. For the specimens containing 3 fiber strands (y3-series), similar α_p_ factors are determined as for the specimens with only one fiber strand. Comparing the two concretes C1 and C2, it becomes clear that the higher-strength concrete leads to somewhat lower α_p_ factors. Furthermore, a general tendency can be seen that with increasing maximum bond flow, the splitting tendency decreases regardless of whether the parameter of concrete or textile is varied. Thus, textile T1bA exhibits a significantly lower splitting tendency factor than textile T1A.

### 3.3. Correlation between the Splitting-Tendency Factors and the Concrete Width per Fiber Strand

**Figure 11:**
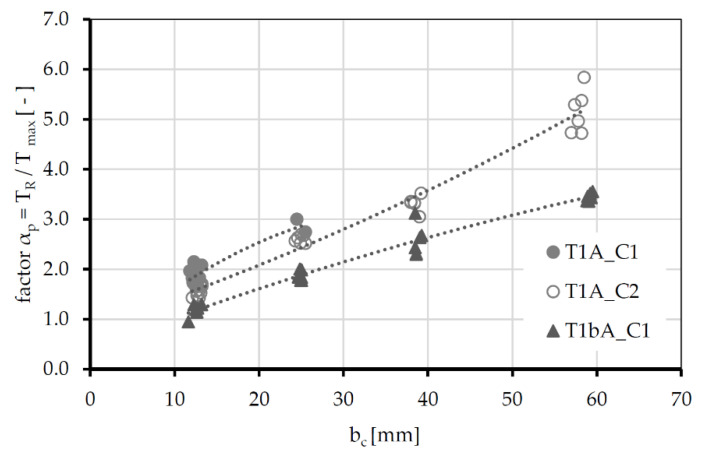
Dependence of the α_p_-factors on the concrete width per fiber strand (b_c_); assumption γ = 1.0 (Equation (1)).

In the further tests, the concrete width per fiber strand increased stepwise (compare Figure 5) and the α_p_ factors were determined. Only tests in which a splitting failure occurs were evaluated. Due to the low bond performance of textile T1B (Figure 9), no investigations on the splitting failure were conducted. In case of textile T2A, only the specimens with b_c_ = 13 mm showed splitting cracks. The maximum bond flow that could be reached was too low to initiate a splitting crack by concrete width b_c_ > 13mm. Thus, an evaluation of the splitting failure was not possible. Figure 11 shows the dependence between the α-factors and the concrete width b_c_.

Independent of concrete type and textile type, the splitting-tendency factors increase with increasing concrete width. However, since factor α_p_ is a textile-specific parameter, which should be similar for each width, an adjustment of the resistance side is necessary. This is achieved by introducing factor γ, which considers the non-uniform stress distribution in the concrete cross-section. The actual stress distribution is replaced by a constant stress block. For textile T1A in combination with concrete C1, no splitting failure of the specimen could be observed above a width of 25 mm, while for concrete C2 even the specimens with b_c_ = 38 mm and 60 mm failed on a splitting crack. This can be explained by the greater maximum bond flow of C2 (see Figure 9) in combination with the more brittle behavior (compare [22]). The splitting tendency thus increases disproportionately to the tensile strength of the concrete, and the failure mode shifts from pull-out without cracking to pull-out with splitting.

### 3.4. Derivation of a Reduction Factor for Determining the Effective Tensile Strength as a Function of the Concrete Width per Fiber Strand

**Figure 12:**
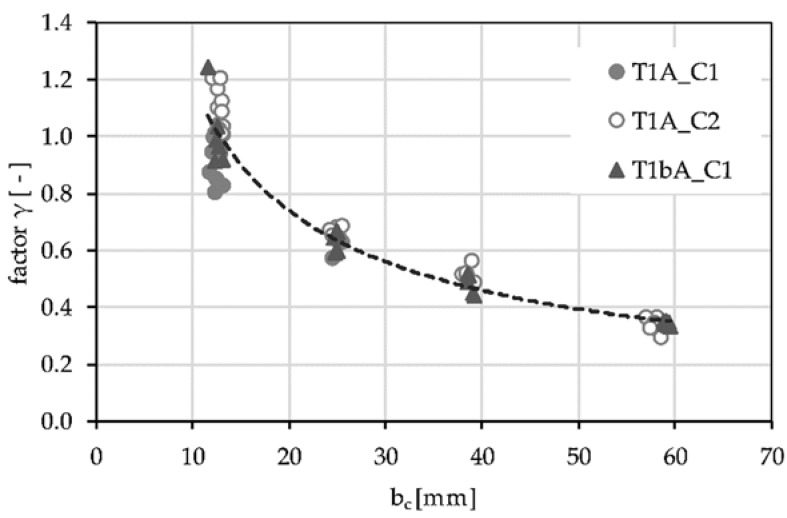
Factor γ for considering the stress distribution in the concrete in relation to the concrete width per fiber strand (b_c_).

For the determination of the representative α_p_ factors, the results of the tests with a concrete width of b_c_ = 13 mm are used (see Figure 10). For textile T1A, α_p_ is set at 1.72 (mean value of all four test series) and for textile T1bA at 1.18. With these constant values, the γ factors for the different concrete widths are determined according to Equation (1). Figure 12 shows the results of this evaluation. A lower γ factor leads to a lower effective concrete tensile strength that can be used to calculate a constant stress block over the entire cross-section.

A uniform tendency is evident for all test series. With increasing mesh width, the concrete strength that can be applied decreases and approaches a limit value of approx. 0.35 at about 60 mm. The relationship between the concrete width per fiber strand and the reduction factor γ can be described by a second-degree function. Considering all results, the function y = 5.7873 * e_0_^−0.687^ describes the relationship best (coefficient of determination R^2^ = 0.95) for e_0_ (is equal to b_c_) between 13 mm and 60 mm. The function is shown as a dashed line in the diagram. In case of e_0_ < 13 mm, the suggested value is 1.0.

**Figure 13:**
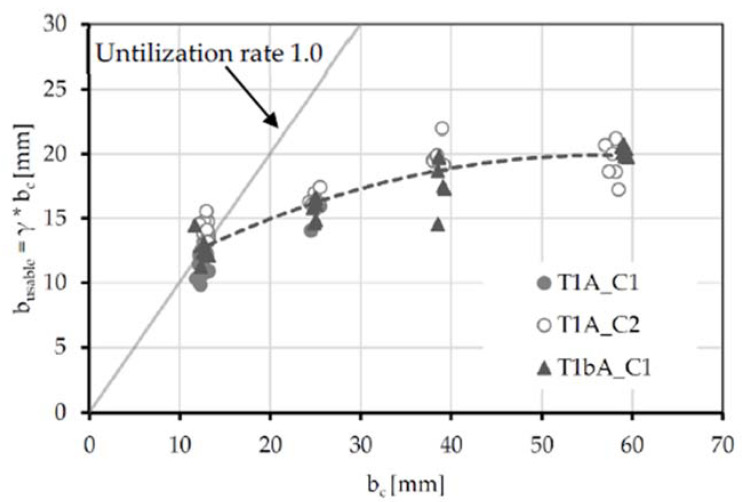
Relationship between the effective usable concrete width (b_usable_) and concrete width per fiber strand (b_c_).

Multiplying the mesh width (b_c_) with the factor γ, an effective usable concrete width (b_usable)_ can be determined. This is plotted in Figure 13 in relation to the concrete width (b_c_). In addition to the test results, the graph for a utilization factor of 1.0 is shown in the diagram.

As the concrete width increases, the effective usable width (b_usable_) increases disproportionately. For example, only b_usable_ = 18 mm of a mesh width of 38 mm can be used. Furthermore, 20 mm represents the limit value for the maximum of effectively usable concrete width. An increase in the mesh width would therefore not lead to an increase in resistance to splitting failure. This behavior is probably due to an initial crack in the boundary zone of the fiber strand, which subsequently grows into the specimen.

## 4. Discussion

Using a concrete of a higher-strength class, the bond forces between textile and matrix can be slightly increased. This provides shorter anchorage lengths in a component. However, a reduction of the crack distance is not to be expected since with increasing concrete tensile strength, the crack load of the concrete increases in addition to the bond strength.

Regarding the splitting-tendency factors (α_p_) of C1 and C2, in combination with textile T1A, it would be appropriate to use different factors for the two fine-grained-concretes. In case of the higher performance C2 concrete, the factor will be reduced to 1.58 (difference 9%). Therefore, the γ factors also have to be reduced, which means the usable tensile strength of the C2 concrete needs to be decreased compared to the one of C1. This supports the thesis of Preinsdorfer [22] that a higher-strength concrete has a relatively lower effective tensile strength due to its greater brittleness. Compared to Preinsdorfer’s results, however, this reduction is less pronounced here.

The calculated reduction curve for increasing concrete width b_c_ is calibrated on two geometrically similar textiles (48 k fiber strand), impregnated with a medium stiff (acrylate) agent. In [7], no reduction factor is calculated, which can be explained by the small mesh sizes (7.2 mm) of the used textile. This confirms the assumption made here that no reduction in tensile strength is required for a concrete width of <12.7 mm. In [22], single-sided pull-out tests on a stiff (epoxy resin) coated 96 k textile with mesh width of 38 mm are conducted. Here, a reduction factor γ = 0.67 by e_0_ = 38 mm is used. In comparison, the relationship presented here results in a decrease to 0.53. The difference can probably be explained by the larger fiber strand width of about 5 mm (here about 3 mm) of the 96 k strand. If the fiber strand has a larger width, the reduction curve shifts upwards because the influence of the yarn on the concrete width increases. Thus, it might be useful to indicate the reduction of the concrete stresses as a function of the yarn width (b_c_/b_yarn_). However, further investigations are necessary to evaluate this statement.

In the presented investigations, the initial splitting crack starts at the fiber strand and develops parallel to the textile layer in the direction of the 90° strand (compare Figure 2). The stresses, causing the initial crack, along the fiber strand (L_0_) are assumed to be constant. Considering the splitting crack failure in an anchorage length, the stress distribution along the fiber strand is not constant. This is a result of the bond-slip curve of the textile. Considering the bond-slip curve, textile T1A reaches the highest bond stresses near the last transverse crack. The bond stresses decrease with increasing anchorage length. Thus, the initial splitting crack starts at the last transverse crack and then continues along the anchorage length (compare [7]). In [6], the maximum loads of double-sided pull-out test did not increase. Thus, the anchorage length was increased from 10 to 40 cm. The local splitting forces at the beginning of the anchorage area exceeded the resistance forces of the concrete. A successive failure always occurs at similar load levels regardless of the anchorage length. Consequently, the question arises of whether the calculated reduction curve can also be used for anchorage and overlapping areas.

The model to predict a cracking along the fiber strand presented here is supported by the assumption that the splitting tendency factor (α_p_) is constant for all tests. Regarding the bond-flow–pull-out way-curves a higher bond flow is connected to a greater pull-out way. The greater the pull-out distance, the more the fiber strand is compressed. The question arises if the compression of the fiber strand influences the splitting tendency factor.

If the tendency of a textile to split needs to be reduced, increasing the mesh size is one way to do this. In this case, the reduction of the effective concrete tensile strength presented here must be considered. Furthermore, increasing the mesh size reduces the degree of reinforcement in the component. As a result, the maximum tensile forces that can be absorbed per textile meter decrease and the crack spacing and crack widths become greater. If the mesh size is doubled, the use of a second textile layer in the component would be a possible solution to keep the degree of reinforcement constant. However, this would also increase the manufacturing costs. In addition, the used concrete must be checked regarding the adaptation of the maximum grain size to the layer spacing. An increase in the risk of longitudinal cracking must also be considered. The splitting tendency of the textile can also be influenced by the shape of the fiber strand and the textile processing. The forces perpendicular and parallel to the textile can be influenced by the cross-sectional fiber strand shape (ratio of height to width of the fiber strand) [19]. A smaller widening of the strand reduces the composite forces and thus the absolute risk of splitting [19]. However, the forces that can be anchored at the same anchoring lengths decrease. The variation of the mesh size to reduce the splitting tendency in the component must therefore be considered with regard to many different parameters. The presented correlation between effective concrete tensile strength and mesh size now allows for a quantitative assessment of the problem.

## 5. Conclusions

The results of the presented investigations show dependence between the concrete strength and the bond performance of textile reinforced concrete. A higher-strength concrete leads to a shift upward of the bond-flow–pull-out distance-curve independent of the textile geometry and the impregnating agent. However, the influence of the concrete is considerably smaller than the influence of different textile configurations. The higher bond forces between the textile and higher-strength concrete also result in a greater risk of splitting cracks.

It is shown that a shift of the failure mode from pullout failure in the lower strength concrete to splitting failure in the higher-strength concrete is possible. This can be explained by the greater brittleness of the higher-strength concrete. A comparison of different textiles in combination with two concretes indicates that the splitting tendency tends to decrease with increasing bond strength, regardless of whether the concrete or textile parameter is varied.

Testing three strands of yarn compared to one strand of yarn only has an insignificant influence on the failure load concerning a splitting failure in a single-sided pull-out test with short anchorage lengths (one mesh width). This leads to the conclusion that the test on a single fiber strand is thus representative of several fiber strands lying next to each other.

When increasing the resistance against splitting failure by increasing the mesh width between the fiber strands, the reduction in the effective concrete tensile strength must be considered. The concrete tensile strength decreases to about 35% when the concrete width is increased from 13 mm to 60 mm. This can be attributed to the stresses in the concrete, which are reduced with increasing distance from the fiber strand. The mechanical model that is presented here includes the factor γ, which transforms the stresses in the concrete to a constant stress block. A polynomial relationship of the second degree is proposed for the determination of this reduction factor in dependency of the mesh width, which describes the test results independent of textile and concrete type. Besides, the correlation can be used to estimate the relative splitting tendency of textiles with different mesh widths.

The question arises of whether the results can be transferred to a varying concrete cover. The concrete cover influences the resistance against a longitudinal crack. Therefore, it has to be considered whether the single-sided pull-out test is suitable. The restoring moments in the specimens presented here disproportionately counteract a longitudinal crack failure.

Additionally, a verification of the transferability of the results to fiber strands with a larger yarn cross-section (e.g., 96 k) and stiffer impregnation materials should be done. Presumably, a scaling factor with respect to fiber strand width and yarn cross-sectional area is required here.

## Figures and Tables

**Table 1:** Properties of the used textiles.

Property/Textile Name	Unit	T1A/T1bA	T1B	T2A
fiber-strand geometry		1/1b	1	2
mesh width	mm × mm	12.7 × 16.0	12.7 × 16.0	25.4 × 25.4
impregnation agent	/	A—acrylat	B—SBR	A—acrylat
covering factor	/	0.68	0.68	0.81
mesh width 0° strands (e_0_)	mm	12.7	12.7	25.4
testing length (L_0_)	mm	16.0	16.0	25.4

**Table 2:** Properties of the fine-grained concrete according to EN 196 [24] and EN 12390-13 [25], *n* = number of results, ±standard derivation.

Property/Concrete	Unity	C1	C2
compressive strength, f_c,cm_	MPa	98.83 ± 3.53 (*n* = 90)	131.98 ± 10.46 (*n* = 78)
flexural tensile strength, f_c,flm_		9.5 ± 1.34 (*n* = 45)	11.72 ± 1.41 (*n* = 39)
modulus of elasticity, E_c,s_		33,081 ± 251 (*n* = 3)	38,602 ± 694 (*n* = 3)
factor δ (f_c,t_/f_c,fl_)	/	0.72	0.85

## Data Availability

The data supporting this study are available from the author on request.

## References

[1] Heid A.-C., Stark A., Will N., Hegger J. (2021). Weitspannende Sandwichelemente mit vorgespannten Textilbetondeckschichten und geschäumter Kernschicht/Widespan sandwich elements with pre-stressed layers made of textile reinforced concrete and foamed PU core. Beton Stahlbetonbau.

[2] Steinbock O., Teworte F., Neis B. (2021). Carbonbeton—Eine neue Verstärkungsmethode für Massivbrücken Teil 3: Planung und Umsetzung der Verstärkungsmaßnahme mit Carbonbeton am Pilotprojekt “Brücken über die Nidda im Zuge der BAB A 648”/Carbon reinforced concrete—An alternative Method for strengthening concrete bridges; Part 3: Design and first experiences of strengthening concrete superstructures with carbon reinforced concrete—Federal highway bridges (A 648) across the river Nidda. Beton Stahlbetonbau.

[3] Kromoser B., Preinstorfer P., Kollegger J. (2019). Building lightweight structures with carbon-fiber-reinforced polymer-reinforced ultra-high-performance concrete: Research approach, construction materials, and conceptual design of three building components. Struct. Concr..

[4] Scheerer S., Zobel R., Müller E., Senckpiel-Peters T., Schmidt A., Curbach M. (2019). Flexural Strengthening of RC Structures with TRC—Experimental Observations, Design Approach and Application. Appl. Sci..

[5] Orlowsky J., Maurer R., Heeke G., Beßling M., Bettin M. (2021). Ressourcenschonende Lärmschutzelemente aus Textilbeton als Alternative für konventionelle Stahlbetonfertigteile/Resource-saving noise protection elements made of textile reinforced concrete as an alternative for conventional precast reinforced concrete elements. Beton Stahlbetonbau.

[6] Schütze E., Curbach M. (2019). Zur experimentellen Charakterisierung des Verbundverhaltens von Carbonbeton mit Spalten als maßgeblichem Versagensmechanismus/Experimental characterisation of the bond behaviour of carbon reinforced concrete with concrete splitting as significant failure mode. Bauingenieur.

[7] Lorenz E. (2014). Endverankerung und Übergreifung Textiler Bewehrungen in Betonmatrices. Ph.D. Thesis.

[8] Bielak J., Spelter A., Will N., Claßen M. (2018). Verankerungsverhalten textiler Bewehrungen in dünnen Betonbauteilen/Anchorage behavior of textile reinforcement in thin concrete components. Beton Stahlbetonbau.

[9] Beßling M., Antons U., Orlowsky J., Hordijk D.A., Luković M. (2018). Potentials of Textile Reinforced Concrete for Lightweight Noise Protection Walls. High Tech Concrete: Where Technology and Engineering Meet.

[10] Banholzer B. (2004). Bond Behaviour of a Multi-Filament Yarn Embedded in a Cementitious Matrix. Ph.D. Thesis.

[11] Lorenz E., Ortlepp R., Parra-Montesinos G.J., Reinhardt H.-W., Naaman A.E. (2012). Bond behavior of textile reinforcements development of a pull-out test and modeling of the respective bond versus slip relation. High Performance Fiber Reinforced Cement Composites 6.

[12] Glowania M., Gries T., Schoene J., Schleser M., Reisgen U. (2011). Innovative Coating Technology for Textile Reinforcements of Concrete Applications. Key Eng. Mater..

[13] Schleser M. (2008). Einsatz Polymerimprägnierter, Alkaliresistenter Glastextilien zur Bewehrung Zementgebundener Matrices. Ph.D. Thesis.

[14] Kulas C. (2013). Zum Tragverhalten getränkter textiler Bewehrungselemente für Betonbauteile. Ph.D. Thesis.

[15] Schneider K., Michel A., Liebscher M., Mechtcherine V. (2018). Verbundverhalten mineralisch gebundener und polymergebundener Bewehrungsstrukturen aus Carbonfasern bei temperaturen bis 500 °C/Bond behavior of mineral-impregnated and polymerimpregnated reinforcement structures made of carbon fibers at temperatures up to 500 °C. Beton Stahlbetonbau.

[16] Lenting M., Orlowsky J. (2019). Einaxiale Zugversuche an textilbewehrten Betonen mit anorganisch getränkten Carbonfasern. Beton Stahlbetonbau.

[17] Krüger M., Reinhardt H.-W., Martin F. (2001). Bond behaviour of textile reinforcement in reinforced and prestressed concrete. Otto Graf J..

[18] Butler M. (2009). Zur Dauerhaftigkeit von Verbundwerkstoffen aus Zementgebundenen Matrices und Alkaliresistenten Glasfaser-Multifilamentgarnen. Ph.D. Thesis.

[19] Preinstorfer P., Kromoser B. (2020). Influence of geometrical parameters on the splitting forces in textile-reinforced concrete. Mater. Struct..

[20] Lorenz E., Ortlepp R., Hausding J., Cherif C. (2011). Effizienzsteigerung von Textilbeton durch Einsatz textiler Bewehrungen nach dem erweiterten Nähwirkverfahren/Efficiency Increase of Textile Reinforced Concrete by Use of Textile Reinforcements from the Extended Warp Knitting Process. Beton Stahlbetonbau.

[21] Tekle B.H., Messerer D., Holschemacher K. (2021). Bond induced concrete splitting failure in textile-reinforced fine-grained concrete. Constr. Build. Mater..

[22] Preinstorfer P. (2019). On the Splitting Behaviour of Textile Reinforced Concrete: Zur Spaltrissbildung von Textilbewehrtem Beton. Ph.D. Thesis.

[23] Ortlepp R. (2007). Untersuchungen zur Verbundverankerung Textilbewehrter Feinbetonverstärkungsschichten für Betonbauteile. Ph.D. Thesis.

[24] Deutsches Institut für Normung e.V. (2016). Methods of Testing Cement—Part 1: Determination of Strength; German Version EN 196-1:2016.

[25] Deutsches Institut für Normung e.V. (2014). Testing Hardened Concrete—Part 13: Determination of Secant Modulus of Elasticity in Compression; German Version EN 12390-13:2013.

[26] Beßling M., Orlowsky J., Derkowski W., Gwoździewicz P., Hojdys Ł., Krajewski P., Pańtak M. (2019). Investigations on the bond behaviour of textiles with various coating in TRC. Proceedings of the Fib Symposium 2019: Concrete—Innovations in Materials, Design and Structures, Krakow, Poland, 27–29 May 2019.

